# Chilaiditi Syndrome: A Case Report, Literature Review, and Proposition of a Novel Management Staging System

**DOI:** 10.7759/cureus.46688

**Published:** 2023-10-08

**Authors:** Yaw Adu, Esere A Nesiama, Arham Siddiqui, Sameer Prakash, Izi Obokhare

**Affiliations:** 1 School of Medicine, Texas Tech University Health Sciences Center, Lubbock, USA; 2 Department of Internal Medicine, University of Texas Health Science Center, San Antonio, USA; 3 Department of Internal Medicine, University of Iowa Hospitals and Clinics, Iowa City, USA; 4 Department of General Surgery, Texas Tech University Health Sciences Center, Amarillo, USA

**Keywords:** chilaiditi's sign, chilaiditi syndrome, colonic interposition, laparoscopy, colorectal surgery

## Abstract

Chilaiditi's sign refers to colonic interposition between the liver and the diaphragm in the right subphrenic space secondary to the relaxation of the suspensory ligaments of the right colic flexure. The diagnosis of Chilaiditi's sign is based on radiological findings with the following three criteria: 1) The right hemidiaphragm must be adequately elevated above the liver by the intestine, 2) the bowel must be distended by air to illustrate pseudo-pneumoperitoneum, and 3) the superior margin of the liver must be depressed below the level of the left hemidiaphragm. In this report, we present the case of a 49-year-old female presenting with signs and symptoms suggestive of Chilaiditi syndrome managed with laparoscopic surgery. We also present a literature review with a summary of previous studies and propose a novel management staging system for this syndrome.

## Introduction

Chilaiditi's sign, also known as the interposition of the colon above the liver, refers to the presence of the colon in the right subphrenic space secondary to the relaxation of the suspensory ligaments of the right colic flexure [1]. This condition was first documented in 1865 by Demetrius Chilaiditi, who reported three cases of patients with incidental benign intra-abdominal free air on routine X-rays [2,3]. The sign is relatively rare, with an incidence of 0.025%-0.28% on abdominal or chest X-rays and 1.18%-2.4% on abdominal CT scans and with a marked 4:1 male-to-female predominance [3]. Chilaiditi's sign is usually asymptomatic but can also present as Chilaiditi syndrome, which presents with respiratory distress, substernal pain, and cardiac arrhythmias [4]. Chilaiditi syndrome typically requires prompt medical intervention, though surgical management may be considered in cases where medical treatment is unsuccessful or if the patient shows evidence of bowel obstruction/ischemia or worsening symptoms [1]. To diagnose Chilaiditi's sign, several criteria must be met [5]: 1) the elevation of the right hemidiaphragm above the liver by the intestine, 2) the distended colon by air to illustrate pseudo-pneumoperitoneum, and 3) the depression of the superior margin of the liver below the level of the left hemidiaphragm.

While there are several cases of Chilaiditi syndrome reported in the literature, there is a paucity of data with regard to its management. In this study, we present a case of Chilaiditi syndrome in a 49-year-old female. Furthermore, we propose a novel staging system for patients with Chilaiditi syndrome based on radiological and clinical findings, with the aim of streamlining future medical management.

## Case presentation

**Figure 1:**
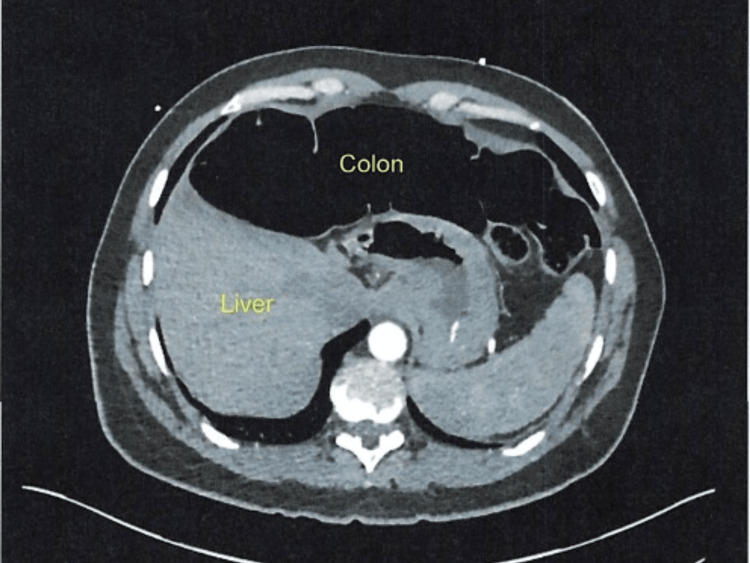
CT of the abdomen revealed a hyperlucent appearance involving the diaphragm diffusely with a lobular appearance, consistent with bowel gas and Chilaiditi syndrome.

A 49-year-old female with no pertinent past medical history presented to the emergency department with a chief complaint of sharp chest pain for 3-4 hours. The pain was localized to the right upper quadrant (RUQ) and epigastric regions, with radiation to the right shoulder and chest. The patient reported an increased frequency of intermittent pain associated with nausea, which was exacerbated by deep breathing and oral intake. The patient also endorsed occasional dysphagia, globus sensation, palpitations, and long-standing chronic constipation. She had no relief with over-the-counter antacids and laxatives for many years and did report a family history of constipation and colonic perforation. Surgical history was significant for appendectomy and cholecystectomy. Physical examination revealed marked abdominal distention and mild epigastric pain with decreased bowel sounds. A CT scan of the abdomen and pelvis revealed a diffusely hyperlucent diaphragm with a lobular appearance, consistent with bowel gas and Chilaiditi syndrome (Figure 1). Follow-up abdominal imaging showed gaseous loops of bowel present throughout the gastrointestinal tract with distention extending down to the rectum. Distended loops of small bowel were noted, with most lying within the left side of the abdomen, necessitating a surgical consultation.

Despite implementing a combination of medical management, lifestyle modifications, the use of multiple laxatives, and changes in diet, her bowel movements continued to be infrequent, occurring every 2-3 weeks. Further evaluation with a colonoscopy, a sitz marker study, and a balloon expulsion test in the absence of anorectal abnormalities excluded other conditions including inflammatory bowel disease, constipation, perforation, and abdominal or pelvic floor dysfunction. After a thorough discussion with the patient regarding the risks and benefits of surgical management, a laparoscopic-assisted subtotal colectomy with ileorectal anastomosis was performed. The patient was later successfully discharged on postoperative day 6.

Postoperatively, the patient's recovery was complicated by the development of a pelvic abscess, which was successfully treated with percutaneous drainage. Subsequently, she reported no nausea or vomiting and was tolerating a diverse diet with an average of 3-5 bowel movements per day.

## Discussion

Upon initial presentation, our patient displayed signs and symptoms consistent with a potential diagnosis of Chilaiditi syndrome including sharp, localized pain in the chest and RUQ of the abdomen accompanied by nausea. The pain was not worse with breathing but may have been triggered by oral intake. Treatment through medical management including oral antacids was unsuccessful, and she required surgical intervention. While our patient's case was successfully managed with surgery, this may not be the case for all patients with this syndrome. Table 1 highlights a case series of varying outcomes in patients managed conservatively and patients who underwent surgical interventions currently present in published literature. Currently, there is no widely accepted standard method for classifying or managing patients with Chilaiditi syndrome. Given the varying responses to treatment, it is crucial that a standardized protocol be developed to aid clinicians with classification and management.

**Table 1:** Case series of Chilaiditi syndrome patient presentations and outcomes after conservative management or surgical intervention. N/A, not available; RUQ, right upper quadrant; GI, gastrointestinal; US, ultrasound; NG, nasogastric

Author	Age	Sex	Presentation	Symptom Length	Radiology	Conservative Management	Surgical Intervention	Outcome
Gad et al. [6]	49	Male	Generalized abdominal pain and cough	48 hours	Chest X-ray and CT of the abdomen	Intravenously administered fluids, cough suppressants, and pain control	N/A	Pain resolved with supportive treatment, and he was stable before discharge. One-year follow-up: asymptomatic and no complications
Bin Waqar et al. [7]	63	Male	Abdominal pain and shortness of breath	8 hours	US of the abdomen and CT of the abdomen	Metoclopramide, enemas, and laxatives	N/A	Aborted initial laparoscopy for conservative management. Gradually responded to conservative measures. Weekly follow-up: asymptomatic and no complications
Melester and Burt [8]	75	Female	Severe RUQ abdominal pain, nausea, vomiting, distension, and hyperactive bowel sounds	4 hours	Roentgenogram	"Symptomatic" treatment	N/A	Two years post treatment: asymptomatic and no complications
Melester and Burt [8]	63	Male	RUQ abdominal pain and epigastric pain	3 days	Roentgenogram and upper GI series	NG decompression	N/A	Three years post treatment: asymptomatic and no complications
de Mesquita et al. [9]	5	Male	Acute RUQ abdominal pain, distension, and respiratory distress	2 weeks	Chest X-ray and US of the abdomen	Fleet enema		One day post treatment: improvement; repeat X-ray showed no subphrenic hypertranslucency. Two-week follow-up: asymptomatic and no complications
Luo et al. [10]	59	Male	Intermittent lower abdominal pain, distension, and defecation difficulties	3 years	Chest X-ray and CT of the abdomen	Conservative treatment was attempted, but the patient was unable to endure abdominal pain, distension, and defecation difficulties	Laparoscopic-assisted right hemicolectomy	Discharged after 14 days of hospitalization with close follow-up. Four-week follow-up period: reported complete resolution of abdominal pain, distension, and defecation difficulties. Nine-month follow-up: asymptomatic and no complications
Yin et al. [4]	58	Female	Generalized migratory intermittent abdominal pain for nine months, RUQ abdominal pain for two days, diarrhea, constipation, and nausea	2 days	CT of the abdomen and pelvis	Stool softeners and analgesics	Pexy of the cecum and ascending colon	One-week follow-up: appendectomy for persistent symptoms. Three-week follow-up: reported complete resolution of abdominal pain with improved regularity of bowel movements. Two-month follow-up: asymptomatic and no complications
Sunkara et al. [11]	56	Female	Generalized abdominal pain and multiple episodes of vomiting	Unknown	CT of the abdomen	N/A	Exploratory laparotomy and right hemicolectomy and end ileostomy on day 7	Two-week follow-up: improvement of symptoms
Acar et al. [12]	54	Male	RUQ abdominal pain, vomiting, nausea, tenderness, and rigidity	24 hours	Chest X-ray and CT of the abdomen	N/A	Exploratory laparotomy for suspected perforation and right hemicolectomy with ileocolic anastomosis due to obstruction	Four-month follow-up: asymptomatic no complications
Shah et al. [13]	55	Male	RUQ abdominal pain, distension, loss of appetite, and constipation	1 year	Chest X-ray and US of the abdomen	N/A	Elective laparotomy with 25 cm resection of the sigmoid colon with anastomosis between the proximal sigmoid and rectum	N/A

We propose a three-stage grading system for Chilaiditi syndrome patients. This system is based on radiological and clinical findings with the goal of simplifying future medical management (Table 2). The proposed grading system includes symptoms such as abdominal pain (which can be diffuse or localized to the RUQ), difficulty tolerating oral intake, chest pain, and vasovagal reactions. The proposed grading system will provide a clear and consistent approach to evaluating and managing Chilaiditi syndrome patients. With this new system, clinicians can make well-informed decisions to improve patient outcomes.

**Table 2:** Proposed staging protocol for patients with Chilaiditi syndrome.

Stage	Symptoms and Severity	Radiological Signs	Management
1	Mild (2-5 times yearly)	Redundant colon with a segment above the liver	Medical
2	Moderate (2-5 times monthly)	Redundant distended colon	Medical and consider surgical consult
3	Severe (2-5 times weekly)	Redundant colon and signs of colonic ischemia	Surgical candidate for subtotal/partial colectomy

**Figure 2:**
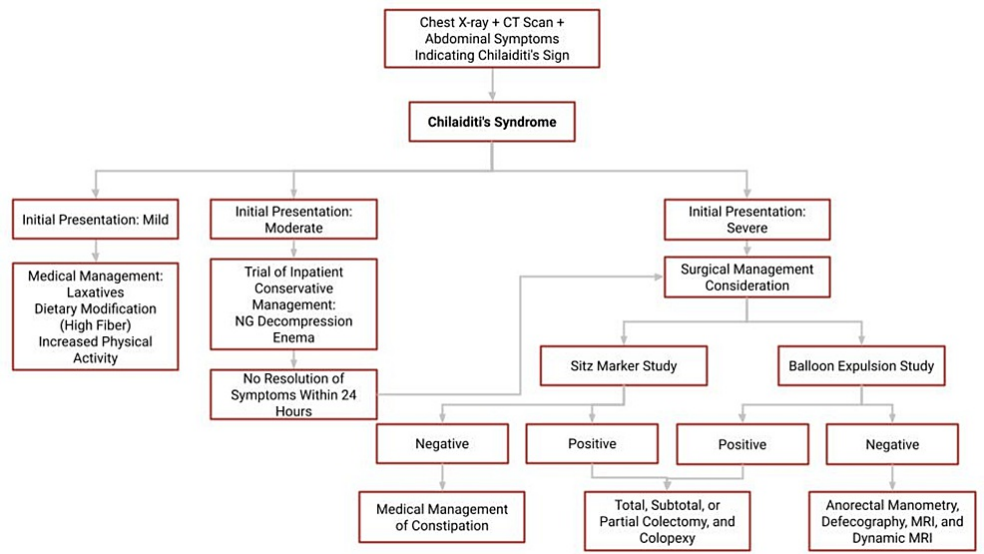
Proposed treatment algorithm to Chilaiditi syndrome to guide optimal management. NG: nasogastric

We also propose the use of our treatment algorithm (Figure 2) to guide management for Chilaiditi syndrome. Following the classifications in Table 2, mild cases of Chilaiditi syndrome (stage 1) should be managed medically with a high-fiber diet, laxatives or stool softeners, and antispasmodics [14]. Additionally, physical activity has been shown to be beneficial by promoting intestinal peristalsis [15]. However, moderate to severe cases (stages 2 and 3) should be worked up further with imaging scans such as gastrografin enemas, CT scans with endoluminal contrast, and colonoscopy. In cases where these imaging studies provide equivocal or normal findings, additional diagnostic tests such as sitz marker studies, balloon expulsion studies, and anal manometry studies should be performed. These studies will help differentiate Chilaiditi syndrome from other benign anorectal maladies. For patients who test positive on these additional diagnostic tests, surgical intervention may be considered as the most suitable treatment option.

## Conclusions

Chilaiditi syndrome is a complex condition that can manifest in various complications, depending on the underlying causes. This report highlights and describes a standardized staging system to guide the medical management of Chilaiditi syndrome patients. This will help ensure that patients receive the most appropriate treatment for their condition and that treatment is tailored to their level of severity. This will ultimately lead to improved outcomes for patients with Chilaiditi syndrome.
